# Nutrition economic evaluation of a probiotic in the prevention of antibiotic-associated diarrhea

**DOI:** 10.3389/fphar.2014.00013

**Published:** 2014-02-17

**Authors:** Irene Lenoir-Wijnkoop, Mark J. C. Nuijten, Joyce Craig, Christopher C. Butler

**Affiliations:** ^1^Department of Pharmaceutical Sciences, University of UtrechtUtrecht, Netherlands; ^2^Scientific Affairs, Danone ResearchPalaiseau, France; ^3^Ars Accessus MedicaAmsterdam, Netherlands; ^4^York Health Economics Consortium Limited, University of YorkYork, UK; ^5^Institute of Primary Care and Public Health, School of Medicine, Cardiff UniversityCardiff, UK

**Keywords:** antibiotic-associated diarrhea, *Clostridium difficile*, hospitalized elderly, probiotics, cost effectiveness, nutrition economics

## Abstract

**Introduction:** Antibiotic-associated diarrhea (AAD) is common and frequently more severe in hospitalized elderly adults. It can lead to increased use of healthcare resources. We estimated the cost-effectiveness of a fermented milk (FM) with probiotic in preventing AAD and in particular *Clostridium difficile*-associated diarrhea (CDAD).

**Methods:** Clinical effectiveness data and cost information were incorporated in a model to estimate the cost impact of administering a FM containing the probiotic *Lactobacillus paracasei ssp paracasei* CNCM I-1518 in a hospital setting. Preventing AAD by the consumption of the probiotic was compared to no preventive strategy.

**Results:** The probiotic intervention to prevent AAD generated estimated mean cost savings of £339 per hospitalized patient over the age of 65 years and treated with antibiotics, compared to no preventive probiotic. Estimated cost savings were sensitive to variation in the incidence of AAD, and to the proportion of patients who develop non-severe/severe AAD. However, probiotics remained cost saving in all sensitivity analyses.

**Conclusion:** Use of the fermented dairy drink containing the probiotic *L. paracasei* CNCM I-1518 to prevent AAD in older hospitalized patients treated with antibiotics could lead to substantial cost savings.

## Introduction

### Etiology and epidemiology of antibiotic-associated diarrhea

Antibiotic-associated diarrhea (AAD) is a form of diarrhea that occurs during or shortly after administration of an antibiotic, and is diagnosed in the absence of other known causes of diarrhea. The rate of occurrence varies among reports, with a range of 1–44%, depending on the population and type of antibiotic (Bergogne-Berezin, 2000; Graul et al., 2009). Adults over the age of 65 years are known to be at the top end of this range (Kyne, 2010; Bauer et al., 2011), and broad spectrum antibiotics impart a greater risk than narrow spectrum, in particular clindamycin, cephalosporins, and fluoroquinolones. One aspect of AAD that compels us to take this problem seriously is the occurrence of *Clostridium difficile*-associated diarrhea (CDAD), also referred to as *C. difficile* infection, in elderly hospitalized patients. CDAD is responsible for around 10–25% of all cases of AAD and it can occur up to 8 weeks after antibiotic therapy (Bartlett and Gerding, 2008).

### Treatment of AAD

AAD is treated by withdrawal of the precipitating antibiotic, avoidance of antiperistaltic agents, rehydration and, if necessary replacement of the provocative agent by a more appropriate antibiotic, which has a lesser risk of induction of diarrhea (Cohen et al., 2010). Clinical resolution is observed in the majority of patients who respond well to these treatments but a small proportion of subjects may develop CDAD. Current guidelines from ESCMID consider pharmacotherapy of an initial episode of CDAD with oral metronidazole or oral vancomycin the mainstays for pharmacological treatment of CDAD (Crobach et al., 2009). English guidelines recommend oral metronidazole for initial treatment in these patients, because it is cheaper than oral vancomycin, and because of the concern that overuse of vancomycin may result in the selection of vancomycin-resistant enterococci (Gerding and Gerding, 2005). In patients with severe *C. difficile* infection, English guidelines recommend initial treatment with oral vancomycin, on the basis of evidence from relatively recent randomized clinical trials (RCTs) that compared vancomycin and metronidazole, showing a lower rate of treatment failure with vancomycin in patients with severe *C. difficile* infection (Louie et al., 2007; Zar et al., 2007; Bouza et al., 2008).

### Recurrent infection

Recurrence and reinfection is common and can occur in up to 50% of the cases, depending on the antibiotics used (Debast et al., 2014). Recurrence of CDAD is a serious and difficult-to-treat problem, impacting on the length and overall cost of hospitalization (Pépin et al., 2005a,b; Fitzpatrick and Barbut, 2012). The antibiotic administered to treat the initial episode may be used for the first recurrence, unless this is metronidazole and the recurrence meets criteria for severe *C. difficile* infection. In second and subsequent recurrences, vancomycin is recommended (Gerding et al., 2008). Fidaxomicin, a novel bactericidal macrocyclic antibiotic (Drekonja et al., 2011; Cornely, 2012) is not part of the routine treatment approaches, although it can be considered in specific situations left to the discretion of the specialists.

### Mortality

Mortality due to *C. difficile* doubled from 1999 to 2004 and continued to rise until 2007. Since then, great effort has been made to ensure prevention or optimized care and a decrease was seen in the UK (Wiegand et al., 2012). However, it is still a significant cause of AAD, posing a considerable financial burden on health service providers in both Europe and the USA (Ghantoji et al., 2010; Wiegand et al., 2012). Evidence suggests that there is an increasing incidence of CDAD in populations previously thought to be at low risk (Benson et al., 2007; Honda and Dubberke, 2014). In recent years there has also been an emergence of new hypervirulent genotypes, in particular the strain BI/NAP1/027, leading to increased incidence of CDAD, more severe disease, higher relapse rates, increased mortality, and greater resistance to fluoroquinolone antibiotics (McDonald et al., 2005; Pépin et al., 2005a,b; Cartman et al., 2010; Barbut et al., 2011). In 2004, a prospective study was conducted at 12 Quebec hospitals to determine the incidence of nosocomial CDAD and its complications (Loo et al., 2005). A total of 1703 patients with 1719 episodes of nosocomial CDAD were identified. The incidence was 22.5 per 1000 admissions. The 30-day attributable mortality rate was 6.9 percent.

### Prevention

English guidelines recommend that healthcare workers wash their hands before and after contact with patients with suspected or confirmed *C. difficile* infection, and that disposable gloves and aprons are used when handling body fluids and caring for CDAD patients (National Audit Office, 2004; Department of Health and Health Protection Agency, 2008). These guidelines also recommend that patients with potentially infective diarrhea should be moved immediately into a single room with en-suite facilities. Prevention primarily revolves around control of antibiotic use, followed by comprehensive infection control procedures once outbreaks occur.

In parallel, other interventions for the prevention of AAD have been explored, including the use of probiotic bacteria (Parkes et al., 2009). Probiotics are defined as “live micro-organisms which, when administered in adequate amounts, confer a health benefit on the host” (FAO/WHO, 2001). A number of meta-analyses of trials with probiotics for prevention of AAD have been performed (Hempel et al., 2012; Ritchie and Romanuk, 2012; Videlock and Cremonini, 2012). We only report here the most recent Cochrane meta-analysis reporting on CDAD (Goldenberg et al., 2013). The primary objectives were to assess the efficacy and safety of probiotics for preventing CDAD in adults and children. Randomized controlled (placebo, alternative prophylaxis, or no treatment control) trials investigating probiotics (any strain, any dose) for prevention of CDAD were considered for inclusion. The analysis (23 trials, 4213 participants) suggests that probiotics significantly reduce the risk by 64%. The incidence of CDAD was 2.0% in the probiotic group compared to 5.5% in the placebo or no treatment control groups (RR 0.36; 95% CI 0.26–0.51). Adverse events were assessed in 26 studies (3964 participants) and this analysis indicates that probiotics reduce the risk of adverse events by 20% (RR 0.80; 95% CI 0.68–0.95). The authors conclude that moderate quality evidence suggests that probiotics are both safe and effective for preventing *C. difficile*-associated diarrhea.

### Economic impact

In 2004, the UK health authorities introduced mandatory reporting of CDAD in people older than 65 years. The majority of cases of CDAD are elderly people with prolonged in-patient stays in a health-care setting. AAD and CDAD have become and remain a serious problem for health care providers, leading to concerns around patient safety, and increased medical treatment costs. The considerable morbidity associated with CDAD results in a high economic burden, with extended length of hospital stays being the main cost driver as patients with CDAD spend on average an extra 7–21 days in hospital, compared with non-infected controls (Campbell et al., 2009; Dubberke and Wertheimer, 2009). Isolation of the patients who develop CDAD can also represent a substantial cost, as well as the closing and cleaning of wards. In addition, there are significant costs associated with treating recurrent infections. Vonberg et al. (2008) reported an average cost to treat CDAD of €33,840 per patient. Furthermore, since a high proportion of CDAD patients are elderly, its economic burden is expected to increase over the coming years as the world's population ages (Kuijper et al., 2006).

Based on the Cochrane report that probiotics can be moderately effective in reducing the incidence of CDAD, we may hypothesize that consumption of a probiotic can reduce the associated increased treatment costs and the extended length of stay in acute health care facilities, if used routinely in elderly patients who receive antibiotics. Hence the objective of this study was to assess the health-economic impact of a preventive nutritional strategy using a fermented milk (FM) with probiotic in hospitalized patients older than 65 years in the UK health care setting.

### Methods

Decision analytic modeling is a well-accepted methodology to estimate cost-effectiveness and inform budgetary impact analysis of an intervention, including not only outcomes from clinical trials, but also data provided by other sources of information on items such as resource utilization, response, and recurrence.

#### Model design

A model was constructed to estimate the cost consequences of a FM containing the probiotic *Lactobacillus paracasei ssp paracasei* CNCM I-1518 as a preventive intervention in the management of AAD, from the perspective of the NHS and in line with current NICE guidelines (NICE, 2008).

**Figure 1:**
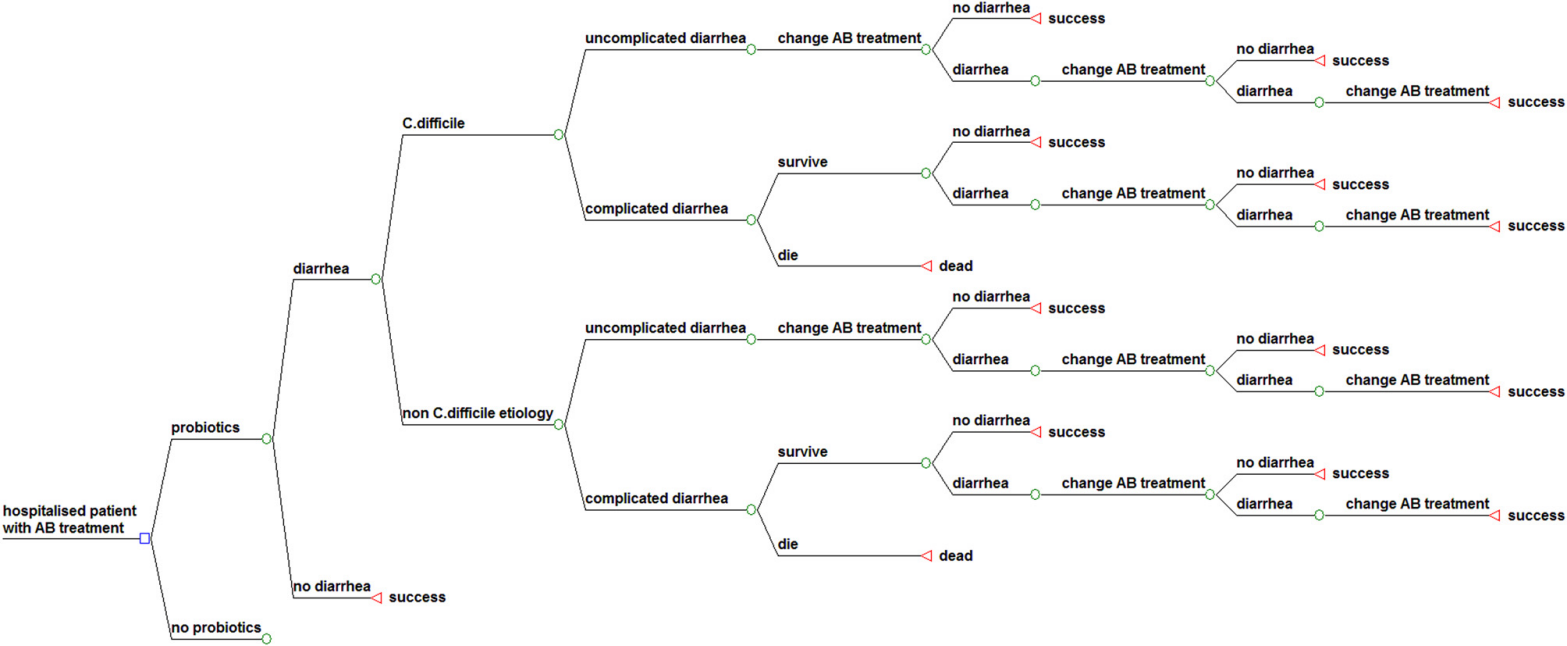
**Structure of the model used in the analysis**.

Figure 1 shows the structure of the model. Our model considers a cohort of elderly hospitalized patients over 65 years of age treated with antibiotics, who may or may not receive probiotics as preventive treatment. The structure for the sub model “probiotics” is identical to the displayed structure of the sub model “no probiotics.” In both groups patients may develop uncomplicated or complicated diarrhea with a related mortality risk. When diarrhea occurs, antibiotic treatment is interrupted or changed, according to the NHS best practice guidance. The patient may respond, leading to cure of the diarrhea, or not respond due to treatment failure, leading to another change of treatment. A proportion of the “cured” patients at any point in the model will suffer a recurrence after an initial response. The structure for the sub model “non-C. diff etiology” is identical to the displayed structure of the sub model “*C. difficile.”*

The follow-up time in this model is until recovery or death during the period of hospitalization, which comprises treatment of AAD, including CDAD and also recurrent infection and complications.

#### Study population and comparison

The study population consists of elderly hospitalized patients over 65 years of age being treated with antibiotics. The study population in our model is based on the study populations in the trials, which provide the clinical input for the model, especially a clinical trial by Hickson (2007), conducted in England. A recent study conducted on behalf of the European Center for Disease Prevention and Control confirmed that age is one of the risk factors for more severe infection (Bauer et al., 2011). The model compares the FM with probiotic as preventive management of AAD, in particular CDAD, with no preventive treatment, according to the UK guidelines for pharmacoeconomic research (NICE, 2008).

#### Clinical outcomes

The model extrapolates the efficacy data from the probiotic clinical trials on AAD (reduction in the rate of CDAD and non-CDAD), and other related clinical events (recurrent diarrhea, complications, and mortality) to calculate overall success rate (proportion of patients fully recovered).

#### Study perspective

This analysis was conducted from the perspective of the NHS. This perspective facilitates comparisons with other UK economic evaluations. Due to the perspective taken, societal or indirect costs were not included in the analysis. The cost assessment is based on the costs for probiotic management of AAD and the cost associated with treatment of complications.

#### Data sources

Data sources included published literature, clinical trials, official price/tariff lists, a Delphi panel study, and national population statistics. Characteristics of clinical events are not country-specific. Hence, data on clinical probabilities were derived from the international literature (Bouza et al., 2008; Lowy et al., 2010; Louie et al., 2011). On the other hand, for costs and information on therapeutic choices, the general recommendation is that country-specific data sources should be used whenever possible (Drummond and McGuire, 2001). Therefore, a country-specific literature search was performed to identify local costs from official price lists in England.

Additional estimates came from a Delphi panel, which allowed expert opinions to be gained on treatment patterns and associated health care utilization in the absence of robust published data. The panel was made up of seven medical specialists practicing in England, who covered the disciplines of internal medicine, gastroenterology, and medical microbiology. All had clinical experience of managing AAD. They answered questions in an *ad-hoc* developed survey on occurrence, treatment patterns, and therapeutic modalities associated with recurrences and complications. The survey instrument was reviewed by the York Health Economic Consortium before use.

#### Probabilities

While some prospective studies on the health benefits of probiotics in AAD have been conducted, our searches identified only one conducted in England (Hickson et al., 2007).

We extrapolated clinical outcome data from this randomized double blind, placebo controlled trial that presented the same setting as assumed for our model. In the trial of 135 hospitalized elderly (mean age 74 years), Hickson and colleagues investigated the efficacy in preventing AAD of a 100 g (97 ml) of a FM containing the probiotic *Lactobacillus paracasei ssp paracasei* CNCM I-1518—formerly referred to as *Lactobacillus casei* DN 114001—given twice a day beginning within 48 h of starting antibiotic therapy and continued until 1 week after the antibiotic treatment finished. The control group received a placebo without probiotics. Of the probiotic group, 12% (7/57) developed AAD compared to 34% (19/56) in the placebo group (*P* = 0.007). None of the patients randomized to the FM with probiotic developed CDAD, while 17% (9/53) in the placebo group developed CDAD (*P* = 0.001). There were no adverse events in the probiotic group.

The incidence of AAD ranges widely, depending on the population and type of antibiotic (Surawicz, 2005; Butler et al., 2012). Our base case analysis is based on a mean incidence of 15%, with sensitivity analysis conducted on a range of 5 and 25%, as indicated in literature (Bergogne-Berezin, 2000).

The risk ratio (RR) for the total population from Hickson's study was 0.35 (12/34), while the most recent Cochrane meta-analysis reports a RR of 0.36 (Goldenberg et al., 2013). We assumed a RR of 0.35 for our base case and conducted a scenario analysis using a RR of 0.36 from the Cochrane meta-analysis.

CDAD accounts for 5–25% of all cases of AAD (Bergogne-Berezin, 2000; Barbut et al., 2007). Our base case assumes that 15% of patients who develop AAD will have CDAD. A sensitivity analysis was based on a range from 10 to 25%. The risk reduction is applied to this incidence data.

The population in the model was stratified by non-severe and severe diarrhea based on frequency estimates from the Delphi panel. CDAD can occur up to 8 weeks after antibiotic therapy (Bartlett and Gerding, 2008). Of the 15% of patients with AAD estimated to have CDAD, 26% was estimated to be severe at initial presentation, with 74% non-severe at the onset, but with 18% of the 74% subsequently becoming severely ill. Of the 85% non-CDAD cases, 12% were estimated to be severe at onset, with among the 88% who were non-severe at the onset 12% becoming severe after diagnosis and treatment (Table 1).

**Table 1:** **Distribution of severe and non-severe CDAD and non-CDAD**.

	**Non-severe (%)**	**Severe (%)**
**Onset of CDAD**	74	26
**Onset of non-CDAD**	88	12
**Progression (1st line) from mild to severe**		
**CDAD**		18
**Non-CDAD**		12

Response to treatment varies significantly between antibiotics, as well as between patients treated with different classes of antibiotics. Clinical resolution of AAD is observed in 80–100% of cases following interruption or change of antibiotic treatment. Delphi panel members provided separate estimates for non-CDAD and CDAD for our model (Table 2). For non-CDAD, the Delphi panel estimated a 73% response rate to first line treatment and an 18% recurrence rate, which we used in the base case.

**Table 2:** **Response and recurrence percentages based on Delphi**.

**CDAD**		
Response 1st line	Response 2nd line	Response 3rd line
69%	51%	47%
**Non-CDAD**		
Response 1st line	Response 2nd line	Response 3rd line
73%	35%	33%
**CDAD**		
Recurrence 1st line	Recurrence 2nd line	Recurrence 3rd line
22%	35%	35%
**Non-CDAD**		
Recurrence 1st line	Recurrence 2nd line	Recurrence 3rd line
18%	11%	6%

We conducted a sensitivity analysis of non-CDAD treatment responses for a response rate of 80% based on previous research findings. We did not include mortality for non-CDAD because we found no relevant published reports of death, and the Delphi panel confirmed this.

For CDAD, the Delphi panel indicated a response rate to first line treatment of 69%, which is used in the base case. As this is lower than the figures reported in literature, we based a sensitivity analysis on an 80% response rate to assess the uncertainty associated with this estimate.

The risk of recurrence after a single episode of CDAD is high. Recurrent CDAD (relapse of diarrhea after initial resolution of symptoms) usually occurs within 1–3 weeks, but has been described up 2 months after the initial episode (Pépin et al., 2006). The Delphi panel estimate of recurrence rate is 22% which lies within the range reported in the literature of 8–50% of patients over 65 years with severe underlying illness having at least a second episode after treatment with metronidazole, and additional antibiotic use (Shannon-Lowe et al., 2010). The estimates by the Delphi panel are used for the base case. A sensitivity analysis for recurrences is based on a range from 8 to 50%.

The Delphi panel estimated a 6.14% mortality rate for CDAD, similar to the 6.9% reported in a study from Quebec (Miller et al., 2002). *C. difficile* causes most of the severe cases of AAD, and therefore in our model, all mortality data were considered to relate to *C. difficile* only and not to other causes of AAD.

#### Costs

Calculations of costing were based on official NHS price lists (British National Formulary, 2009; Department of Health Reference Costs, 2009/10; PSSRU Unit Costs of Health and Social Care, 2010) in combination with the information on treatment modalities as provided by the Delphi panel. Additional expenses included the cost of the probiotic product.

The cost of the FM containing the probiotic *Lactobacillus paracasei ssp paracasei* CNCM I-1518 (Actimel®) is based on twice-daily intake of one bottle of 100 g and cost of GBP 0.33 × per bottle. There was no additional cost for the comparator «no preventive treatment». The clinical effectiveness rates have been described in the section “*probabilities*” under Methods. The cost of hospitalization was based on length of stay and per diem costs. The length of stay was derived from the Delphi panel and *per diem* costs were based on the official NHS price lists

The Delphi panel provided estimates on treatment pathways and drug prescription: all patients with non-CDAD and CDAD are treated in line with the NICE guidelines. The cost of drug treatment included in our model is based on the average of the indications provided by the Delphi panel members and included the options of stop of antibiotic treatment and switch of medication.

Management of AAD frequently increases the length of hospital stays. The additional length of stay and other related resource utilization were derived from the Delphi panel. The Delphi estimates on treatment modalities for an episode of non-severe, severe and recurrent AAD, and associated costs are provided in Tables 3–5, respectively. Table 6 lists the unit costs.

**Table 3:** **Costs in GBP based on Delphi for AAD (non-severe)**.

**Non-severe**	**CDAD**	**Non-CDAD**
**1st line**		
Hospitalization	£2268	£1614
LOS (in days)	4.3	3.1
Consultations	£141	£74
Specialist (number of visits)	2.4	1.4
Other^*^ (number of visits)	2.3	1.6
Medication	£35	£1
Other	£159	£113
Total	£2602	£1802
**2nd line**		
Hospitalization	£ 2867	£ 796
LOS (in days)	5.4	1.5
Consultations (same as 1st line)	£141	£74
Medication	£96	£5
Total	£3104	£875
**3rd line**		
Hospitalization	£2513	£627
LOS (in days)	4.8	1.2
Consultations (same as 1st line)	£141	£74
Medication	£154	£1
Total	£2808	£702

*LOS, length of stay*.

* *Microbiology, GP, junior doctor, district nurse, pharmacist, consultant, gastro-enterologist*.

**Table 4:** **Costs in GBP based on Delphi for AAD (severe)**.

**Severe**	**CDAD**	**Non-CDAD**
**1st line**		
Hospitalization	£5688	£2897
LOS (in days)	10.7	5.5
Consultations	£192	£147
Specialist (number of visits)	4.1	5.4
Other^*^ (number of visits)	4.0	5.3
Medication	£93	£2
Other	£319	£242
Total	£6292	£3287
**2nd line**		
Hospitalization	£5953	£3441
LOS (in days)	11.2	6.6
Consultations (same as 1st line)	£192	£147
Medication	£90	£30
Total	£6236	£3617
**3rd line**		
Hospitalization	£4702	£3074
LOS (in days)	8.9	5.9
Consultations (same as 1st line)	£192	£147
Medication	£216	£15
Total	£5110	£3235

LOS, length of stay.

* Microbiology, GP, junior doctor, district nurse, pharmacist, consultant, gastro-enterologist.

**Table 5:** **Costs in GBP based on Delphi for AAD (recurrence)**.

	**Non-severe**		**Severe**	
	**CDAD £**	**Non-CDAD £**	**CDAD £**	**Non-CDAD £**
1st line recurrence	3079	871	6237	3610
2nd line recurrence	3127	881	6286	3633
3rd line recurrence	3236	874	6416	3806

**Table 6:** **Unit costs in GBP**.

		**Cost £**	**Fraction**	**Fee £**
Specialist		36.5	0.5	73
GP		47		47
Microbiology		43.8	0.3	146
Junior doctors		6.9	0.3	
District nurse		10		10
Pharmacist		45	1	45
Consultant		43.8	0.3	146
Gastro-enterologist		43.8	0.3	146
Per diem	General ward	530		
	Single ward	479		
Full blood count		3.29		
Urea and E		5.72		
FBC		5.72		
CRP		1.73		
Kioch		5.72		
WBC		5.72		
LFT/UE		5.72		
X-abdomen		169		
Flex symo		253		
Microsopy		11.81		
Culture		11.81		
Toxin		11.81		
Storhs c/S		11.81		
Storh WBS		11.81		
MC+S		11.81		
C. diff PCR		11.81		

## Results

The base case shows that when the FM with probiotic is used, £243 is saved on average per case treated with antibiotics by preventing non-CDAD, whereas £96 is saved on average per case treated with antibiotics through preventing CDAD (Table 7).

**Table 7:** **Sensitivity analyses**.

			**Cost savings £**
Incidence AAD^*^	Base	0.15	339
	Low	0.05	101
	High	0.25	576
Proportion CDAD^*^	Base	0.15	339
	Low	0.1	319
	High	0.25	379
Proportion non-severe^*^	Base	0.74	339
	Low	0	638
	High	1	283
Risk reduction^*^	Base	0.35	339
	Cochrane	0.36	332
Response	Base: CDAD	0.69	339
	Non-CDAD	0.71	
	Literature^***^	0.8	318
Recurrence^**^	Base: CDAD	0.22	339
	Non-CDAD	0.17	
	Low: CDAD	0.08	304
	Non-CDAD	0.05	
	High: CDAD	0.5	407
	Non-CDAD	0.42	

* Based on literature.

** Based on Delphi.

*** Upper limit based on literature; no need for lower range, because this is not realistic. If literature reports between 80 and 100% response.

Although the cost impact for CDAD is much higher, the total cost savings for CDAD are lower than for non-CDAD because its represents only 15% of the total AAD cases that occur in older hospitalized patients receiving antibiotics. The overall results for both non-CDAD and CDAD show that the mean cost of managing a case of AAD without using a probiotic preventive strategy is £555 per patient, whereas the mean cost is £216 per patient when the probiotic preventive strategy is used. The probiotic intervention therefore leads to potential total mean cost savings of £339 per hospitalized patient over 65 years treated with antibiotics, regardless of whether he/she develops AAD.

### Sensitivity analyses

Sensitivity analyses on the incidence of AAD, the proportion of CDAD, the proportion of patients who develop non-severe/severe AAD, risk reduction based on the meta-analyses, and estimated response rates and recurrence (Table 8) show that the cost savings are sensitive to the incidence of AAD (£100–£576) and to the proportion non-severe/severe patients (£283–£638). A sensitivity analysis based on risk ratio from the most recent Cochrane meta-analysis showed an estimated cost savings of £332 for all forms of AAD. The outcomes remain cost saving in all the sensitivity analyses undertaken. These analyses indicate that the FM with probiotic is cost-effective by preventing AAD in comparison to no preventive strategy.

**Table 8:** **Base-case (costs in GBP)**.

	**Costs probiotics £**	**Other £**	**Total £**	**Survival**
**CDAD**				
Probiotics	9.24	58.64	67.88	0.9995
No probiotics	0.00	163.98	163.98	0.9986
Difference			96.10	−0.0009
**Non-CDAD**				
Probiotics	9.24	139.07	148.31	1
No probiotics	0.00	391.02	391.02	1
Difference			242.71	0
**Total**				
Probiotics	9.24	206.95	216.19	0.9999
No probiotics	0.00	555.00	555.00	0.9998
Difference			338.81	−0.0001

## Discussion

In our study, we developed a decision analytic core model to estimate the health economic impact of a FM containing the probiotic *L. paracasei* CNCM I-1518 for the prevention of AAD in patients hospitalized aged over 65 years, in England. The rational for performing this health economic analysis is that the incidence of AAD has a high impact on the health care resources used. Therefore, interventions to prevent AAD may have an important health economic impact.

This analysis has some limitations. First of all, our Delphi panel made specific estimates for England based on experiences from their daily practice. As treatment with antibiotics and the success rates are often country-specific, this may explain some differences between the estimates from our Delphi panel and findings from international research. For example, the Delphi panel estimated a 73% response rate for treatment of AAD, which is lower than estimates from empirical studies. Sensitivity analyses performed on the input variables to assess the level of uncertainty associated with response and recurrences showed that the cost savings estimates are sensitive to the incidence of AAD, and the proportion of patients with severe AAD. However, the direction of outcomes of the model remained consistent in all sensitivity analyses, which confirms the robustness of the model.

Secondly, one may question the relevance of considering the cost-effectiveness of a preventive probiotic strategy in AAD, given the on-going debate on the clinical efficacy of probiotics in this indication and the heterogeneous outcomes. A large multicenter trial, recently published in Lancet found no differences between the treatment group and the control group in the incidence of AAD (Allen et al., 2013). However, this may partly be explained by the choice of the probiotics used, as well as by the low event rate (Daneman, 2013), in particular of CDAD. This notwithstanding, *C. difficile* infection is the leading cause of diarrhea in industrialized countries (Jones et al., 2013) and the Office for National Statistics states that: “Over 80 per cent of *C. difficile* infections are in people aged over 65 years” (www.ons.gov.uk).

This raises the issue of the generalizability from the strict clinical evidence toward health care in real life conditions, comparable to those reported by Maziade (2013) in an open prospective study over a 7-years period, including a total of 31,832 hospitalized patients receiving antibiotics (Maziade et al., 2013).

Finally, a restriction with regards to external validity is that the chosen probiotics lactobacilli and bifidobacteria in the study by Allen are only two types of non-pathogenic bacteria, and therefore do not represent daily practice, where much more types of probiotic strains are used. As a consequence, the results from a meta-analysis may have a lower internal validity, but on the other hand have a higher external validity because of the inclusion of various available probiotic strains. Therefore, the results from the meta-analysis provide a more profound clinical input for a health economic analysis.

FM with probiotic, taken as part of the usual meals, also has nutritional benefits (Radavelli-Bagatini et al., 2013), which can be of particular importance to the elderly hospitalized population. The RCT that informed our analysis did not report any adverse effects related to the dairy product. When adverse effects do occur with probiotics, they are generally mild and resolve on withdrawal of the probiotic (Allen et al., 2010; Hempel et al., 2011).

The current health economic analysis is based on a patient-based cost-effectiveness model. As a consequence the following clinical and economic issues are not captured in the analysis: resistance of the antibiotic as such, costs of person-to person transmission of CDAD, costs associated with isolation of patient, and closing and cleaning of wards, and transmission.

## Conclusion

This analysis shows the interest of taking into consideration the cost-impact of use of a fermented dairy drink containing the probiotic *Lactobacillus paracasei ssp paracasei* CNCM I-1518 to prevent AAD, and in particular CDAD compared to no preventive treatment with probiotics in hospitalized elderly receiving antibiotics, as this approach potentially allows for considerable cost savings to the NHS.

## Author contributions

All authors contributed to the manuscript: Irene Lenoir-Wijnkoop and Mark J. C. Nuijten initiated the study and developed the study design. Mark J. C. Nuijten and Joyce Craig developed and quality assured the model, Christopher C. Butler provided input into the clinical aspects, the analysis plan and validated the assumptions. All were involved in literature review and interpretation of the results and finalizing of the manuscript, written and coordinated by Irene Lenoir-Wijnkoop.

### Conflict of interest statement

Irene Lenoir-Wijnkoop is an employee of Danone Research. Mark J. C. Nuijten, Joyce Craig, and Christopher C. Butler have had no financial relationships with any organizations that might have an interest in the submitted work in the previous three 3 years.
